# When Cisgender, Heterosexual Men Feel Attracted to Transgender Women: Sexuality-Norm Violations Lead to Compensatory Anti-Gay Prejudice

**DOI:** 10.1080/00918369.2021.1938467

**Published:** 2021-06-29

**Authors:** Keon West, Martha Lucia Borras-Guevara

**Affiliations:** Equalab, Department of Psychology, Goldsmiths, University of London, London, UK

**Keywords:** Sexuality gender norms, traditional gender norms, attitudes toward gay men, prejudice

## Abstract

Cisgender, heterosexual men’s adherence to gender norms and prejudice against sexual minorities increases after observing sexuality-based gender norm violations of others (i.e., non-normative sexual attractions). No research to date has investigated whether similar effects occur after experiencing sexuality-based gender norm violations of the self. This study investigated the effects of one such norm violation—attraction to transgender women—on adherence to gender roles and attitudes toward gay men. Photographs of female models were shown to cisgender, heterosexual men (*N* = 135, *M* age ± *S.D*. = 28.12 ± 8.81) who rated their attractiveness. Half the participants were informed (accurately) that the models were transgender women (transgender condition). Other participants were not offered this information, leaving them to assume the models were cisgender. All participants then reported their support for traditional gender norms and attitudes toward gay men. As expected, participants in the transgender condition reported less positive attitudes toward gay men, an effect mediated by increased support for traditional gender norms, and only present when the participants had rated the women as highly attractive. These results suggest a strategy to compensate for gender norm violations to reestablish men’s masculinity.

Despite meaningful improvements on legislation for LGBT rights across the world, equal marriage in more than 21 countries, and public policy tackling discrimination and bullying against sexual and gender minorities (Bachmann & Gooch, 2018), prejudice against the LGBT community remains a serious and pervasive problem. In fact, in the last 4 years (2015–2019), the United Kingdom has shown a significant increase in the prevalence of hate crime toward these minority groups, with crime reports on the grounds of sexuality and gender identity rising by 27% and 45%, respectively (National LGBT Survey, Summary Report, 2019).

Although anyone can potentially commit a hate crime against a sexual or gender minority (Williams & Tregidga, 2013), most studies concur that perpetrators of these crimes are usually, young, cisgender (those individuals who were classified as men when they were born, and whose gender identity matches that classification), heterosexual, White men (Chakraborti, Garland, & Hardy, 2014; Iganski, Smith, Dixon, & Bargen, 2011; Tebbe, Moradi, & Ege, 2014; Williams & Tregidga, 2013). Trying to explain this, researchers have suggested that the high incidence of young, cisgender, straight men as anti-LGBT aggressors may reflect men’s strategy to defend and preserve gender norms or the gender/sex binary (Bosson, Weaver, Caswell & Burnaford, 2011; Glick, Gangl, Gibb, Klumpner, & Weinberg, 2007; Morgenroth & Ryan, 2020; Parrott, 2009; Talley & Bettencourt, 2008). These norms have been defined for men as any behavior or experience that reinforces a man’s status as a “real man” (Bosson et al., 2011). Globally, cultural perceptions around the idea of a white manhood converge to a prototypical strong, dominant, masculine, cisgender, heterosexual, male character (Bosson & Vandello, 2011; Morgenroth & Ryan, 2020). Further, the status of a “real man” is hard to attain in most cultures, and once achieved has to be constantly protected through active demonstrations and avoidance of any masculinity-threatening behaviors (Bosson, Vandello, Burnaford, Weaver, & Wasti, 2009; Bosson, Weaver, Caswell, & Burnaford 2012; Gilmore, 1990). Consequently, when a man’s male role is under threat, active, aggressive actions may be expected as a means to restore their manhood to spectators (Bosson et al., 2009; Morgenroth & Ryan, 2020).

It should be pointed out that although masculinity perceptions among men who identify as white and heterosexual may be similar in some contextual levels (e.g., being dominant), they may also differ at other levels. For instance, men’s interpretation of sexual activities (e.g., sex between men) can be completely different depending on the population being studied (Silva, 2017): Men in rural areas have been found to interpret this behavior as a mechanism to strengthen their marriages and hence their heterosexuality, while White men in cities view it as an activity that goes against what it means to be straight (Silva, 2017).

There are many possible ways for a man to inadvertently threaten his masculine status. These can be collectively referred to as gender-norm violations. These violations include those related to occupation (e.g., working in a profession interpreted as feminine, or earning less than one’s wife; Schneider, 2012), appearance (e.g., wearing pink, having long hair, or being slender; Cash & Brown, 1989), personality (e.g., behaving in a way that is perceived as effeminate; Glick et al., 2007) and sexuality (e.g., non-normative sexual attractions; Mahaffey, Bryan, & Hutchison, 2005). It is worth noting that these violations are not objective. Rather, it is well established that gender is a socially constructed concept in Western societies, where men and women are perceived as opposite genders reflecting biological sex and men are encouraged to have and enforce essentialist views on sexuality (Bosson & Michniewicz, 2013; Falomir‐Pichastor & Hegarty, 2014; Morgenroth & Ryan, 2020).

Much prior research has suggested that men’s violation of gender norms in any of these domains can lead to compensatory behaviors that reestablish/maintain men’s traditional privileged masculinity. In terms of the occupation domain, Bittman, England, Sayer, Folbre, and Matheson (2003) found that heterosexual couples who deviate from the normative income standard (e.g., couples in which women earn more money than men), restore this imbalance by having a more traditional division of work at home. Schneider (2012) found similar results for men who had a “woman’s job,” such as a nurse or secretary. Concerning personality gender norm violations, Willer (2005) found that men displayed stronger support for banning same-sex marriage when they had been informed that their personality test scores were more typical to that of a woman. In line with this, Glick et al. (2007) found that, when heterosexual male participants were told that their personalities were more feminine than average, they showed significantly less positive attitudes toward feminine gay men, compared to participants who had been told their personalities were average on masculinity (see also Kilianski, 2003; Talley & Bettencourt, 2008 for similar findings). Altogether, these results have been interpreted as men’s defensive strategy against the stereotyped, undesirable traits that they want to deny about themselves (Govorun, Fuegen, & Payne, 2006).

Despite this wealth of prior research, only a few studies have focused on male gender norm violations related to sexuality. Additionally, this research has studied the sexuality of others but not of the self. For instance, Mahaffey et al. (2005) had men participants observe nude and seminude photos of heterosexual and gay couples (e.g., lesbian and gay). Their findings showed that participants’ antigay attitudes only predicted negative physiological reactions when shown pictures of male gay couples. These results give further support to the argument that when male gender norms have been violated, men react defensively to restore their masculinity. However, they do not yet identify the consequences of sexuality-based male gender norm violations of the self, or whether adherence to gender norms mediated the outcomes.

Attractions to same sex individuals are known to be the first indications of being gay, lesbian, or bisexual (Savin-Williams & Diamond, 2000). In fact, mainstream perceptions of heterosexuality stress that men should exclusively feel attraction and desire for women (Ward, 2015). Therefore, it is not surprising that anecdotal and qualitative research has hinted at the effects relating to sexuality-based gender norm violations of the self (e.g., being attracted to the same sex). In fact, in multiple trials for the assault or murder of trans women, perpetrators have attempted to use a “trans-panic defense” to explain their actions (C. Lee & Kwan, 2014, p. 77). This argument asserts that the heterosexual male defendants had sex (usually oral or anal sex) with a transgender woman they were attracted to under the assumption that she was a *cisgender* woman, and that the realization that she was a transgender woman had made them feel gay, and had felt like a “theft of their heterosexuality” (C. Lee & Kwan, 2014, p. 111).

For instance, in 2002, Michael Magidson and Jose Merel beat Gwen Araujo to death (Lee, 2006). During their trial these two men argued that once they discovered Gwen was a transgender woman, with whom they had anal sex, this had led to trans panic and hence her murder. The defense team, even claimed that due to the effectiveness of Gwen’s feminizing hormone therapy, she had tricked Juan and Michael into believing she was a cisgender woman when indeed she was a man (Lee, 2006; Szymanski, 2005). Relatedly, other research has also shown that individuals categorize transgender women as a subset of gay men, rather than of women (Gazzola & Morrison, 2014). A heterosexual man with such an opinion (i.e., a belief that transgender women are really gay men) would therefore experience attraction to a transgender woman as a threat to his sexual orientation. Adding to this reasoning, straight men who think they will receive unwanted sexual interest from bisexual and gay men have been found to be more prejudiced against these two groups (Pirlott & Neuberg, 2014). It is worth noting that this current research is certainly not endorsing the view that transgender women are gay men, or that attraction to them is in any objective sense a gender-norm violation. However, one must recognize the prior research showing that a non-trivial proportion of people do hold such perceptions, and it is important to investigate how these perceptions may affect responses to gender- and sexuality-based minorities.

This prior evidence suggests at a specific causal chain; when self-identified cisgender, heterosexual men find themselves attracted to transgender women, some may perceive it as a threat to their masculinity and heterosexuality (C. Lee & Kwan, 2014; Mahaffey et al., 2005; Morgenroth & Ryan, 2020). Upon experiencing that threat, these men should engage in behaviors meant to restore their masculinity and distance themselves from gay men (Glick et al., 2007; Kilianski, 2003; Morgenroth & Ryan, 2020; Talley & Bettencourt, 2008). These behaviors could include a greater adherence to traditional male role norms and more negative attitudes toward gay men. Although these effects have previously been suggested, no research to date has directly tested whether they do in fact occur.

## Current research

The main objective of this research was to demonstrate that, for at least some cisgender, heterosexual men, experiencing attraction to transgender women can lead to defensive, compensatory responses including increased adherence to male role norms and anti-gay prejudice. We expected straight, male participants who reported feeling attracted to transgender women to show more anti-gay attitudes as a way to distance themselves from being labeled as gay. We also hypothesized that this effect would be more pronounced for men who had reported higher attractiveness ratings of pictures of trans women (a moderated effect). Furthermore, in line with prior research on other types of gender-norm violations, we expected this effect to be mediated by an increase in adherence to traditional gender norms.

## Methods

Research protocols were approved by the relevant university Ethics Committee. Written informed consent was obtained from all participants in the study. All data were collected and stored in compliance with the UK’s Data Protection Act.

### Participants and design

A-priori power analyses were conducted using G*Power 3 (Faul, Erdfelder, Buchner, & Lang, 2009). Assuming a medium effect size for the hypothesized interaction of condition (transgender women vs. assumed cisgender women) x level of attraction on anti-gay attitudes, and using the following parameters—effect size (*f*) = .25, α = .05, power = .80—it was found that 128 participants would be sufficient for adequate power. Extra participants were recruited to compensate for possible attrition. One hundred and thirty-five men were recruited in London using word of mouth and informational posters, distributed by a research assistant. Participation in this study was voluntary and no monetary incentives were given to participants. To be eligible, men had to identify themselves as both cisgender and heterosexual. Though we acknowledge that human sexuality is complex and multifaceted, for the purpose of this study we sought only to recruit men who identified themselves as simply heterosexual and cisgender. Self-categorization is a widely used and widely accepted method of identifying such participants (Bosson et al., 2009; Gazzola & Morrison, 2014; Parrott, 2009; Parrott & Zeichner, 2005, 2008; West & Cowell, 2015). Accordingly, participants were asked what their gender was, what their sexual orientation was, and if they identified as a sexual or gender minority.

Participants ranged in age from 18 to 71 (*M*= 28.12, *SD *= 8.81), and all described themselves as white. We used a between-participants design, with participants being randomly assigned to one of two conditions: one in which they would be informed that the pictures shown to them were of transgender women and the other where no information was given to them (transgender women condition versus cisgender women condition).

### Stimuli used

A total of three different pictures were shown to all participants in a randomized order. Pictures were chosen from online open access magazines (e.g., steemit.com; sources provided in the Appendix). The images used in this study were of transgender women (*M* age = 33.2, *SD *= 2.5) of different ethnicities (e.g., Filipino American, Thai and White American) who were already publicly identified as transgender women, and already involved in an attractiveness-based industry (e.g., modeling). These pictures were hence in the public domain. All pictures were selected to evoke high attractiveness ratings and a panel of pilot participants (*N*= 30; university students), all agreed that the pictures shown to them were attractive on a scale from 1 to 5 (1 being not attractive at all and 5 being very attractive), and all assumed that they were cisgender women until otherwise informed.

### Procedure

Participants were informed that they were to take part in a study about human sexuality, and explained that they would be shown pictures of women, which they would rate in attractiveness on a scale from 1 to 5 (1 being not attractive at all, and 5 being very attractive). After participants had performed the attractiveness ratings of all pictures, half of them were informed (accurately) that the pictures belonged to models who were transgender women (*transgender* condition). The other half were only told that the pictures belonged to models, leaving participants to assume (as those in the pilot study had assumed) that they were cisgender women (*cisgender* condition). Subsequently, all participants answered questions related to their level of agreement to statements about gender, social norms and their attitudes toward gay people.

Participants gender role norms were measured through six statements related to the anti-feminism subscale of male gender roles (Thompson & Pleck, 1986). These statements consisted of negative, strong emotions related to women’s typical behaviors and attitudes. Participants were asked how much they disagreed (1) or agreed (7) with each statement—e.g., “Unless he was really desperate, I would probably advise a man to keep looking rather than accept a job as a secretary”) (Thompson & Pleck, 1986). Higher values indicated greater support for traditional gender norms. The six items related to gender role norms formed a reliable scale (α = .88). This scale has been widely used as its original sample size was considerable (*N*= 400) and as it has consistently shown a moderate-to-high reliability (α = .76). To measure attitudes toward gay men we used Wright, Aron, McLaughlin-Volpe, and Ropp (1997) 7-point, 6-item semantic differential scale in which participants responded to a set of opposing descriptions to their responses to gay men: cold–warm, positive–negative (reversed), friendly–hostile (reversed), suspicious–trusting, respectful–contempt (reversed), admiration–disgust (reversed). This scale has been widely used to assess attitudes toward a number of groups (Turner, Hewstone, & Voci, 2007; Turner, Hewstone, Voci, & Vonofakou, 2008) including gay men (West & Hewstone, 2012), and has displayed good reliability in its original study (α = .86), as well as good internal reliability in the current study (α = .93). The average of the six items for each of the scales was used in subsequent statistical analyses. All statements used to measure support for traditional gender norms and attitudes toward gay men are shown in the Appendix. When participants were finished, they were debriefed about the nature and purpose of the study.

## Results

### Preliminary analyses

As expected, participants’ attractiveness ratings of the three pictures were on average high (*M*= 4.52, *SD *= 0.72), and did not differ between conditions *t* (1, 125) = 0.41, *p*= .68, *d*= .07, CI [−.202, .308]). Additionally, there was no significant difference in participant’s age between the transgender (*M =* 28.34, *SD =* 9.45) and cisgender condition (*M*= 28.04, *SD *= 8.57), *t* (1, 125) = .18, *p*= .85, *d*= .03, CI [−2.87, 3.46]).

### Differences between conditions

An independent sample t-test was conducted with conditions (*transgender* vs. *cisgender*) as the independent variable and support for traditional gender roles and attitudes toward gay men as dependent variables. This test revealed that support for traditional gender roles was significantly higher for men who had been informed that the pictures shown to them were of transgender women (transgender condition: *M*= 4.46, *SD *= 1.82) than participants who were not given any information about the pictures (cisgender condition) (*M*= 3.45, *SD *= 1.32), *t* (1, 119) = 3.54, *p*= .001, *d*= .58, CI [.44, 1.58]). Likewise, positive attitudes toward gay men were significantly lower for men in the transgender condition (*M*= 3.84, *SD *= 1.94), compared to men in the cisgender condition (*M*= 4.52, *SD *= 1.35), *t* (1, 123) = −2.29, *p*= .024, *d*= .63, CI [−1.26, −.09]).

### Moderation effects

We ran two moderation analyses, via PROCESS macros, Model 1, with pre-standardized variables, 95% confidence intervals (CIs) and 1,000 bias-corrected bootstrap samples. These moderation analyses used condition as the independent variable and attractiveness ratings of women’s pictures was included as the moderator. Either traditional gender roles or attitudes toward gay men were included as the dependent variable.

**Figure 1.**
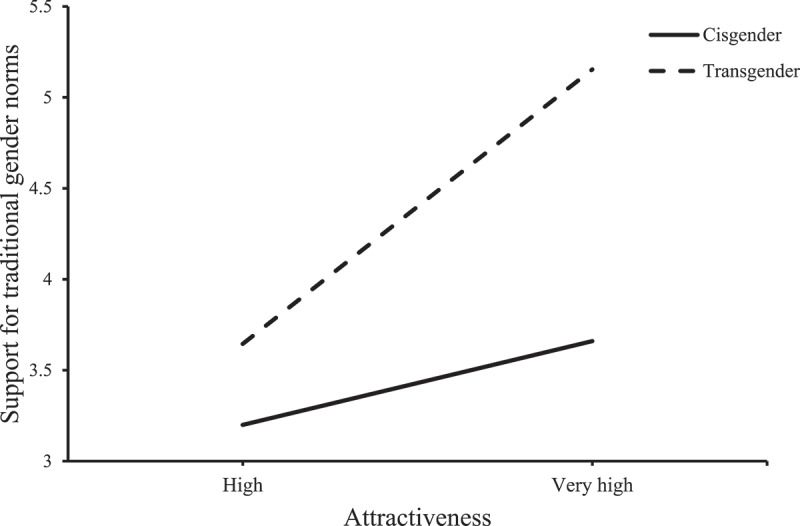
Attractiveness ratings moderation effect of condition on support for traditional gender roles.

In terms of mean traditional gender roles, the model was significant (*F* (3, 117 = 13.69, *p*< .001, *R^2 ^*= .26). Condition (*β *= −2.28, *SE =* 1.01, *t = −2*.07, *p*= .04, CI [−4.46, −.09]) and attractiveness ratings (*β *= .28, *SE =* .14, *t =* 2.04, *p*= .043, CI [.008, .551]) had a significant main effect on traditional gender roles. As expected, the perceived attractiveness of the women in the photos positively predicted adherence to traditional gender roles, but this relationship only applied to the transgender condition, not the cisgender condition. Most important for our central hypotheses, attractiveness ratings moderated the effect of condition on support for traditional gender roles (*β *= .64, *SE =* .24, *t =* 2.65, *p*= .0089, CI [.162, 1.112]. When attractiveness was high (i.e., 5), participants in the transgender condition reported stronger endorsement of traditional gender norms than did participants in the cisgender condition (*β =* .91, *SE *= .19, *t* = 4.71, *p* < .001, CI [.527, 1.29]). Nonetheless, when attractiveness was lower (i.e., 4), there was no significant difference between participants in the cisgender and transgender condition (*β =* .27, *SE *= .20, *t* = 1.31, *p* = .19, CI [−.137, .678]); see Figure 1.

**Figure 2.**
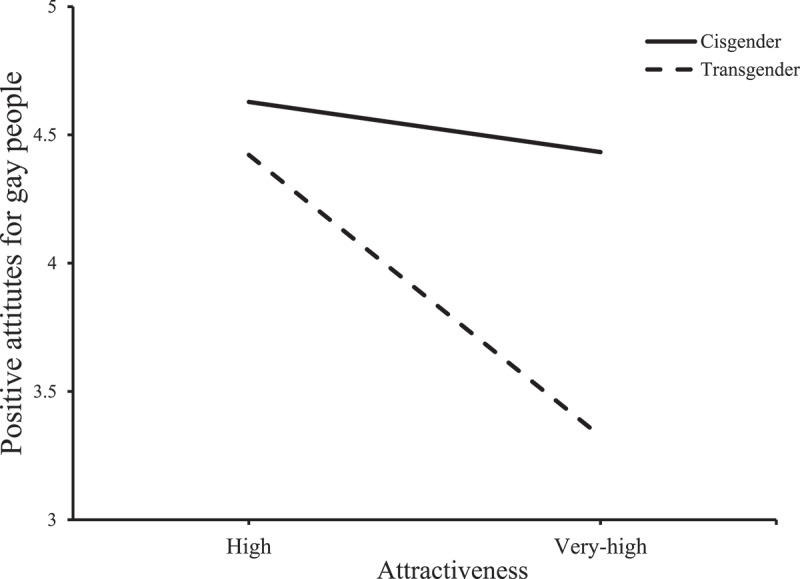
Attractiveness ratings moderation effect of condition on positive attitudes toward gay people.

Concerning mean positive attitudes toward gay men, the model was significant (*F* (3, 121 = 5.62, *p*= .0012, *R^2^ *= .12). Condition showed a trend to significance (*β *= 2.03, *SE =* 1.15, *t =* 1.77, *p*= .079, CI [−.24, 4.29]) on positive attitudes toward gay people. There were no significant main effects of attractiveness rating (*β *= −.12, *SE =* .15, *t = −.7*9, *p*= .43, CI [−.41, .18]). However, more relevant to our hypotheses, attractiveness ratings moderated the effect of condition on positive attitudes toward gay people (*β *= −.54, *SE =* .25, *t = −2*.15, *p*= .033, CI[−1.03, −.043]). When attractiveness was high (i.e., 5), participants in the transgender condition reported lower positive attitudes than did participants in the cisgender condition (*β = −.6*6, *SE *= .21, *t*= −3.20, *p*= .0017, CI [−1.07, −.26]). However, when attractiveness was lower (i.e., 4), there was no difference between participants in the transgender and cisgender conditions (*β = −.1*2, *SE *= .22, *t*= −.57, *p*> .05, CI [−.55, .30]); see Figure 2.

### Moderated mediation

**Figure 3.**
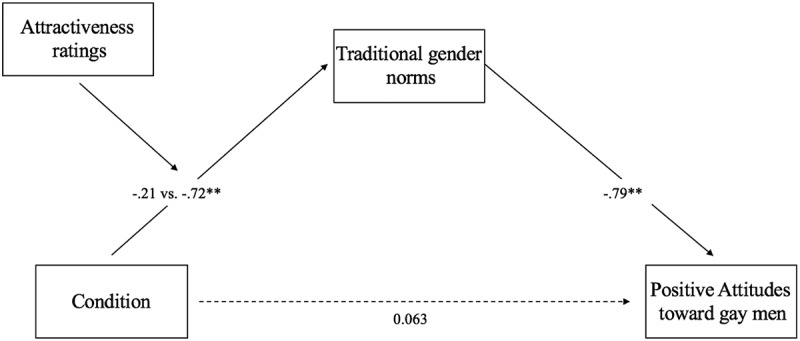
Moderated mediation model of the relationship between condition and positive attitudes toward gay men via traditional gender norms, moderated by attractiveness ratings.

We ran a moderated mediation analysis via PROCESS macros, Model 7, with pre-standardized variables, 95% confidence intervals (CIs), and 1,000 bias-corrected bootstrap samples. This model included positive attitudes toward gay people as the dependent variable, condition (transgender vs. cisgender) as the independent variable, support for traditional gender role norms as mediator and attractiveness ratings of women’s pictures as a moderator (Figure 3). Traditional gender norms mediated the effect of condition on attitudes toward gay people (*β = −.7*9, *SE *= −.06, *t*= −13.06, *p <* .001, CI [−.91, −.67]). When participants showed greater support for traditional gender norms, their positive attitudes toward gay people were lower. This mediation effects was only significant when male participants had rated the pictures shown to them as highly attractive (*β = −.7*2, *SE *= .18, CI [−1.08, −.36]) and not when they had rated them as attractive (*β = −.2*1, *SE *= .15, CI [−.53, .05]).

Note (1): ** = *p* <.001.

## Discussion

The goals of the current study were fourfold. First, we extended past work on the effect of sexuality-based gender norm violations on attitudes toward gay men by examining violations pertaining to the self, while using an experimental paradigm that reflects a real-life scenario: men finding themselves attracted to a woman whose cis- vs. transgender identity is unknown to them. Second, unlike past studies we measured both participants’ support for traditional gender norms and attitudes toward gay men, which have been previously linked to heightened feelings of gender threat in men. Third, we studied the moderating role of attractiveness ratings in the relationship between condition (transgender vs. cisgender) and either positive attitudes toward gay men or support for traditional gender norms. Fourth, we tested a possible mechanism for negative attitudes toward gay men that included support for traditional gender norms as a mediator between condition (transgender vs. cisgender) and attractiveness ratings. In what follows, we summarize our findings and where appropriate we comment on remaining questions, possible directions for future studies, and the implications of these results.

### Moderation analyses

#### Less positive attitudes toward gay people

Negative attitudes toward sexual and gender minorities have been explained in terms of heterosexual men’s strategy to protect and maintain gender norms (Bosson et al., 2011; Glick et al., 2007; Parrott, 2009; Talley & Bettencourt, 2008). In the present study, white men with the transgender condition, who had given higher ratings of attractiveness to transgender women pictures, showed less positive attitudes toward gay people, compared to white men with the cisgender condition. These results may reflect men’s attempt to detach themselves from the anomalous, prejudiced category (*homosexuality*), and to restore the status of their own privileged, standard category (*heterosexuality*). According to this interpretation, men may try to make up for their own deviance by becoming more stringent when it comes to gender sexuality norm departures. This reasoning aligns with findings from Willer (2005), Glick et al. (2007) and Talley and Bettencourt (2008) in which men showed negative attitudes and behaviors toward gay men when their gender personality norms were put under threat. Similarly, these results parallel those found in Swim, Ferguson, and Hyers (1999), where they show that heterosexual people use distancing behaviors as a way of distinguishing themselves from gay people, and that this distancing behavior is related to prejudice.

Men who have been aggressive toward transgender women have argued in their defense that having a sexual relationship with a transgender woman had felt like a “theft of [their] heterosexuality” (C. Lee & Kwan, 2014, p. 111) and that they had been *made gay* (Lee, 2006). Therefore, the fact that white men in the transgender condition of our study showed less positive attitudes toward gay people than white men in the cisgender condition may reflect that sexuality gender norm violations elicit rejection to the specific category that participants believed they could be associated with *Gay men*. In keeping with this interpretation, Gazzola and Morrison (2014) found that men tend to classify transgender women as a subset of gay men. Alternatively, our findings about the influence of sexuality norm deviance on attitudes toward gay men, could equate to effects translating to attitudes toward other sexual and gender minority groups, including any outgroup member who threatens the “heteronormative” value system (Broussard & Warner, 2019).

Although we inquired about attitudes toward gay men when having a manipulation that involved transgender stimuli, we used this design because our main interest was in men’s defensive reactions. Our results, however, highlight the possibility that men’s defensive reactions may be targeted to sexual and gender minorities in general and are not target specific. Future research should look at this issue, comparing defensive strategies against different sexual and gender minority groups, by specifically assessing attitudes that also include the stimuli’s subject as well (e.g., attitudes toward transgender people when having transgender stimuli).

#### Greater support for traditional gender norms

Abundant research has found greater endorsement of traditional gender roles when men face a gender norm threat (Bittman et al., 2003; Black & Stevenson, 1984; Herek, 1988; Schneider, 2012). In line with this, we found that white men who had violated a sexuality gender norm (like participants in the transgender condition), who had given high attractiveness ratings to the women’s pictures showed greater support for traditional gender norms. Stereotype gender roles are known to be highly prescriptive (Eagly & Wood, 1999), hence the greater support for traditional gender roles in individuals who feel they have violated sexuality gender norms may reflect participants way to reestablish their own masculinity and avoid gender misclassification by endorsing feminine roles for women and masculine-dominant roles for men (Bosson et al., 2009; Rudman & Fairchild, 2004).

### Moderated mediation

The link between support for traditional gender norms and attitudes toward gay people has frequently been suggested in psychological studies (Alden & Parker, 2005). However, only a few studies have focused on studying this relationship empirically (Alden & Parker, 2005; Cotten-Huston & Bradley, 2000). To our knowledge, we are the first to explore and confirm the mediation effect of support for traditional gender norms on the relationship between sexuality-norm violations of the self and attitudes toward gay men. In line with previous research (Cotten-Huston & Bradley, 2000; Ficarrotto, 1990; Kite & Deux, 1987; Kurdek, 1988; Macdonald & Games, 1974; Thompson, Grisanti, & Pleck, 1985), we found that participants in the transgender condition were more accepting of gender social norms and less positive about gay men, but only if they had found the women’s pictures to be more attractive.

As such, our findings showed that threatening the heterosexual status of white men reduces positive attitudes toward gay men by increasing their endorsement of traditional gender norms. We suggest that these results are evidence of men’s strategy to avoid and distance themselves from a deviant, prejudiced category. Importantly, it should be noted that although these findings are the result of an in-lab experimentation, they reflect a real-life scenario. In general, men can feel attracted to any women regardless of their cis- vs. transgender identity. Most of the time men assume that a woman is cisgender unless told otherwise (as in our experiment). In times when the killing of transgender women are justified by claiming that perpetrators had been lain to and had been made gay due to this, the revelation that an attractive woman is a transgender woman is exactly the kind of scenario this research was meant to investigate. Future research could take this work further, focusing on men’s behavioral and other responses to transgender women in these scenarios.

## Limitations

The current research focused on white, cisgender, heterosexual men’s defensive reactions when a sexuality-gender norm violation of the self had taken place: these responses included adherence to male role norms and anti-gay prejudice. Although this study has a number of strengths that should be considered including the use of a novel and ecologically valid methodology, there are also some limitations leading to promising future research. All the participants in this study were British, making it unclear whether the effects found here apply to other social contexts. Prior research suggests that support for traditional gender norms varies cross-culturally (Wood & Eagly, 2002). In fact, the same sexual activities can have different interpretations across populations and circumstances (Silva, 2017). For instance, Silva (2017) found that rural men who engage in sex with other men, interpreted their actions as a way to reinforce their heterosexuality being less of a threat to their marriages, a perspective that would be challenged in a different context. Therefore, future research could investigate whether our findings occur, the conditions under which it happens, and to what extent the model presented here replicates in other societies.

Additionally, we need to acknowledge that our sample was in its entirety composed of white men, which limits the generalizability of our findings. Previous literature has shown that white and black men from the same country, usually endorse the same male role norms (Mahalik, Pierre, & Wan, 2006); however, the extent to which our results generalize to a wider population should be the focus of future studies.

Further, it is important to highlight that the intersection of race and gender identity has a critical effect on the aggression risk for trans women. In fact, transgender women of color are disproportionately affected by violence and harassment compared to white transgender women (Bukowski et al., 2019). This could mean that men’s strategy to compensate for sexuality gender norm violations related to transgender women of color may be heightened (e.g., even less positive attitudes toward gay men and greater support for traditional gender norms) or different from what we found. This should be the focus of future studies.

Interestingly, transgender people do not exclusively identify themselves as men or women (Doan, Quadlin, & Powell, 2019). Straight, cisgender men may place more importance to the biological (anatomical) construct of sex rather than the socially constructed concept of gender. It is therefore important to understand whether our findings transcend the gender binary in future research.

Critics of this study may suggest that the findings presented here (significantly less positive attitudes against gay people), are simply due to the effect of participants feeling deceived after the manipulation. Although this is certainly a possible explanation for the rise in prejudice against any group, it does not necessarily explain the higher support from traditional gender norms found here. Additionally, several researchers have found evidence in support of deceptive experimental techniques not inducing negative perceptions in participants’ responses (Christensen, 1988; Sharpe, Adair, & Roese, 1992).

## Implications

Given the discrimination against transgender women, a minority group that has been the target of violence and prejudice across a range of cultures all over the world (Bandini & Maggi, 2014), it is important to acknowledge how the results presented here help explain why they occur, and perhaps lead to ways to reduce cissexism (assuming that the gender identity and behavior of cisgender people are more legitimate than those of trans people) in our society. Accordingly, our findings suggest that interventions designed to change gender norms, perhaps men’s sexual orientation perceptions based on gender identity assigned at birth, could help reduce the already high violence and prejudice experienced by trans women. Further, previous literature has shown that children as young as 26 months of age are aware of gender role stereotypes (Weinraub et al., 1984); therefore, it is likely that interventions would be more efficient if done during the early stages of a child’s development. It is also important to note the relevance of experimental studies that resemble naturally occurring scenarios (like this one), in its contribution to understanding causes of negative behaviors. Our results have the potential of being used in psychotherapy with transgender women who have experienced sexual prejudice from straight men. Understanding why men behave in such a way against them may help lessen the impact this could have in their lives (Koken, Bimbi, & Parsons, 2009).

## Conclusion

Consistent with our predictions from gender norm threat theory (Herek, 1986, 1988; Kilianski, 2003; Kimmel, 1997; Kite & Whitley, 1996), we found that the effect of men’s sexuality-based gender norm violations of the self, parallel results of those related to sexuality norm violations of others. In addition, in line with previous literature, we found that attractiveness ratings moderated the effect of conditions (cisgender vs. transgender) on support for traditional gender roles and positive attitudes toward gay people. Further, a moderated mediation analysis revealed that participants who reported greater support for traditional gender norms, had less positive attitudes toward gay people. This mediation effect was significant only when male participants had rated the pictures shown to them as highly attractive. These findings suggest a strategy to restore men’s manhood by distancing themselves from a deviant sexual orientation category (*homosexuality*).

## Data Availability

The data that support the findings of this study are openly available in Open Science Foundation at https://osf.io/5sfyb/?view_only=4961abecd0594a8598dc8bdbaa969abc

## Appendix

Stimuli and sources:

Source for picture 1 (Geena Rocero): https://bit.ly/39AdsDu

Source for picture 2 (Claudia Charriez): https://bit.ly/39CXWXq

Source for picture 3 (Treechada Petcharat) https://bit.ly/2xpBhzB

Attitudes against gay men:

Respondents were asked to rate their feelings toward gay men on six bipolar evaluative dimensions (based on Wright et al., 1997):

- warm/cold
- negative/positive
- friendly/hostile
- suspicious/trusting
- respect/contempt
- admiration/disgust

Support for traditional gender norms:

1. Unless he was really desperate, I would probably advise a man to keep looking rather than accept a job as a secretary.
2. It bothers me when a man does something that I consider “feminine.”
3. A man whose hobbies are cooking, sewing, and going to the ballet probably wouldn’t appeal to me.
4. It is a bit embarrassing for a man to have a job that is usually filled by a woman.
5. If I heard about a man who was a hairdresser and a gourmet cook, I might wonder how masculine he was.
6. I think it is extremely good for a boy to be taught to cook, sew, clean the house, and take care of young children.
7. I might find it a little silly or embarrassing if a male friend of mine cried over a sad love scene in a movie.
